# A Host Factor GPNMB Restricts Porcine Circovirus Type 2 (PCV2) Replication and Interacts With PCV2 ORF5 Protein

**DOI:** 10.3389/fmicb.2018.03295

**Published:** 2019-01-08

**Authors:** Kangkang Guo, Lei Xu, Mengmeng Wu, Yufeng Hou, Yanfen Jiang, Jiangman Lv, Panpan Xu, Zhixin Fan, Ruiqi Zhang, Fushan Xing, Yanming Zhang

**Affiliations:** ^1^College of Veterinary Medicine, Northwest A&F University, Yangling, China; ^2^College of Life Sciences, Northwest A&F University, Yangling, China

**Keywords:** porcine circovirus type 2 (PCV2), transmembrane glycoprotein NMB (GPNMB), open reading frame 5 (ORF5), Cyclin A, virus-host interaction

## Abstract

Porcine circovirus type 2 (PCV2) is the infectious agent of postweaning multisystemic wasting syndrome (PMWS). The recently discovered open reading frame 5 (ORF5) in PCV2 genome encodes a non-structural protein. Previous study revealed that ORF5 protein inhibits cell proliferation and may interact with host transmembrane glycoprotein NMB (GPNMB). However, whether the GPNMB affects PCV2 replication and the underlying molecular mechanisms are still unknown. In this study, the transcriptome maps of PCV2-infected and ORF5-transfected porcine alveolar macrophages 3D4/2 (PAM) cells were profiled. The GPNMB gene was down-regulated in PCV2-infected and ORF5-transfected PAMs. By using glutathione S-transferase (GST) pull-down, co-immunoprecipitation (co-IP) and confocal microscopy approaches, we convincingly showed that PCV2 ORF5 protein interacts with GPNMB. Furthermore, by utilizing lentivirus mediated overexpression or knockdown approach, we showed that the cellular GPNMB significantly inhibits PCV2 replication and ORF5 expression. Moreover, GPNMB overexpressing leads to an increased Cyclin A expression and a reduced S phase, whereas GPNMB knockdown causes a decreased Cyclin A expression and a prolonged S phase. In conclusion, we identified a novel host factor GPNMB that interacts with PCV2 ORF5 protein and restricts PCV2 replication.

## Introduction

Porcine circovirus type 2 (PCV2) is the leading cause of postweaning multisystemic wasting syndrome (PMWS) which brings tremendous economic losses to swine industry (Darwich et al., 2004; Opriessnig et al., 2007). PCV2 is a non-enveloped, single-stranded, closed-circular DNA virus, belonging to the genus *Circovirus* in the family *Circoviridae* with 1767 or 1768 nucleotides (Meehan et al., 1997). Eleven PCV2 open reading frames (ORFs) have been predicted with six have been well characterized (Ellis et al., 1998; Li et al., 2018). The ORF1 (nucleotides 51–995) gene encodes the Rep protein to initiate replication (Mankertz et al., 1998). The ORF2-encoded Cap protein is the only structural protein and is an immune-associated protein (Nawagitgul et al., 2000). The ORF3 protein has been identified in 2005 as an inducer of apoptosis (Liu et al., 2005). The ORF4 protein is not essential for viral replication but involved in host cell apoptosis inhibition (He et al., 2013). The ORF5 was characterized by our group and has been shown localizes to the endoplasmic reticulum (ER) and induces ER stress (Lv et al., 2015). Notably, it is reported that PCV2 ORF5 does not affect host cell apoptosis but inhibits host cell proliferation via the prolongation of S phase (Lv et al., 2015).

The yeast two-hybrid assay has showed five host proteins interact with ORF5, including transmembrane glycoprotein NMB (GPNMB), cytochrome P450 1A1 (CYP1A1), 14-3-3 protein beta/alpha (YWHAB), zinc finger protein511 isoform X2 (ZNF511) and serine/arginine-rich splicing factor 3 (SRSF3; Yu et al., 2008; Lv et al., 2015). GPNMB is a type I transmembrane protein containing an N-terminal signal peptide, an integrin-binding (RGD) motif and a polycystic kidney disease (PKD) domain in extracellular domain (ECD), a single pass transmembrane domain and a 53 amino acid (AA) cytoplasmic tail (Selim, 2009; Singh et al., 2010). Previous studies have showed the GPNMB is involved in various physiological and pathological processes, including immune system activation, cell proliferation, angiogenesis, tissue-repair, especially the invasion and metastasis of malignant tumors (Rose et al., 2010; Oyewumi et al., 2016). Emerging studies have generated a more complex picture regarding the expression of GPNMB in various cancer progression, including lung cancer, ovarian cancer, stomach cancer and breast cancer (Singh et al., 2010; Zhou et al., 2012; Maric et al., 2013).

Viral replication is strictly relied on host cellular physiological processes. Accumulating evidence demonstrated that the subversion of host cell cycle is a common mechanism employed by virus to facilitate its replication (Swanton and Jones, 2001; He et al., 2002; Laichalk and Thorley-Lawson, 2005; Grey et al., 2010; Balistreri et al., 2016). As one indispensable physiological process, cell cycle contains a series of consecutive biochemical switches allowing the DNA replication of cell genome at the S-phase, subsequently generating daughter cells (G1 and G2-phase) via the equal division during mitosis (M-phase) and quiescent cells are referred as being in G0-phase (Harper and Brooks, 2005). The binding of Cyclins and Cyclin-dependent kinases (CDKs) is required for the entry into the cell cycle phases (Morgan, 1995). It has been reported that the activation of p53 pathway induced by PCV2 infection causes the S phase accumulation, which provides favorable conditions for efficient viral replication (Xu et al., 2016).

Although the GPNMB has been reported interact with PCV2 ORF5 by yeast two-hybrid assay (Lv et al., 2015), whether the GPNMB affects PCV2 replication and the underlying molecular mechanisms are still unknown. In this study, we convincedly demonstrated that PCV2 ORF5 protein interacts with cellular GPNMB, which was also identified as a novel cellular factor that inhibits PCV2 replication. In addition, we also revealed that GPNMB positively regulates Cyclin A expression and triggers a higher proportion of cells to enter S-phase. Taken together, our study identified a novel host factor GPNMB that interacts with PCV2 ORF5 and restricts PCV2 replication and the molecular mechanisms was further deciphered.

## Materials and Methods

### Cells and Virus

Porcine alveolar macrophages 3D4/2 (PAMs) (ATCC: CRL-2845) were grown in RPMI 1640 medium (Solarbio, China) with 10% fetal bovine serum (FBS) (Gibco, United Kingdom). Human embryonic kidney (HEK293T) and porcine kidney (PK-15) cells were cultured in Dulbecco’s modified Eagle’s medium (DMEM) (Solarbio, China) with 10% FBS. PCV2 Yangling strain (wtPCV2) was isolated from one pig with naturally occurring PMWS and propagated in PAMs or PK-15 cells (Tang et al., 2011).

### Plasmids Construction

pEGFP-ORF5 and pEGFP-C1 plasmid was stored in laboratory of Veterinary Public Health and Food Safety, Northwest A&F University, Yangling, China (Lv et al., 2015). PCV2 ORF5 gene was amplified and inserted into pEGFP-C1 with Flag tags both at its N and C terminus to generate Flag-ORF5. ORF5 was inserted into pGEX-6P-1 to generate GST-ORF5. GPNMB gene was amplified from PAMs cDNA and cloned into pCDH-CMV-MCS-EF1 with Flag tags both at its N and C terminus to generate Flag-GPNMB. GPNMB was inserted into pDsRed-N1 to generate pDsRed-GPNMB. Three pairs of shRNAs targeting to GPNMB gene and a random sequence negative control naming shN were predicted^1^ and designed. The fragments were cloned into pCDH-U6-MCS-EF1-GreenPuro after annealing to generate GPNMB-sh1, GPNMB-sh2, GPNMB-sh3 and shN lentivectors. All primers used were listed in Supplementary Table S1. All plasmids were verified by sequencing.

### Infection or Transfection

PAMs cultured in six well plates were incubated with PCV2 at a multiplicity of infection (MOI) of 1.0 for 1 h, then replaced with fresh RPMI 1640 medium with 2% FBS. All eukaryotic expression plasmids were transfected into cells cultured in six plates using the TurboFect Transfection Reagent (Thermo Fisher Scientific, #R0531) according to the manufacturer’s instructions.

### RNA Extraction, Library Construction and High-Throughput Sequencing

To investigate the cellular response after PCV2 infection, PAM cells were infected with PCV2 Yangling strain. PAM cells were infected at a MOI of 1 and then replaced with fresh RPMI 1640 medium containing 2% FBS. Meanwhile, to elucidate the role of ORF5 played in PCV2-induced cellular response, PAMs cells were transfected with pEGFP-ORF5 plasmid. Total RNA was isolated from PAMs (control), pEGFP-ORF5 transfected and PCV2 infected PAMs using Trizol (TAKARA, China) following the manufacturer’s instruction. The RNA samples were submitted to Gene Denovo Co. (Guangzhou, China) for high-throughput sequence (each group was triplicate) and the analysis of the data was also conducted by this company. The RNA samples were assessed for integrity, quality and quantity. 5 μg mRNA was purified from total RNA, adsorbed to oligo (dT) magnetic beads and converted to cDNA used for PCR following size selection by agarose gel electrophoresis (AGE). The obtained RNA was fragmented and subjected to reverse transcription by the random N6 primer. Next, double-stranded DNA was synthesized from the cDNA. Then fill-in and phosphorylate the synthesized double-stranded DNA at the 5’ end. The 3’ end forms a sticky end and then ligate with an adapter that have a bubbling T shape at the 3’ end. The ligation product is PCR amplified by specific primers. The PCR product is heat-denatured into a single strand, and a single-stranded DNA is cyclized with a bridge primer to obtain a single-stranded circular DNA library. The Illumina sequencing was conducted with Illumina Cluster Station and Illumina HiSeq 2000 System. Q20 and Q30 were used to assess the accuracy of base sequences. The software SOAPnuke was used to evaluate the quality of sequencing reads^2^ (Cock et al., 2010). The raw sequencing data were filtered as follows: (i) remove the reads with adapter sequences. (ii) remove the reads with ambiguous bases (N) > 5%. (iii) remove reads with low quality (defined as reads with > 20% of the bases with quality scores < 10).

### Identification of Differentially Expressed Genes (DEGs)

To insure clean and high-quality reads, all raw samples for sequencing were purified by Illumina pipeline, which were mapped to the reference sequences in the UniGene database of *Sus scrofa* (Li et al., 2009). The Reads Per Kilobase Million (RPKM) values, eliminating the interference of gene length and quantity, were used to estimate each gene expression level (Mortazavi et al., 2008). The false discovery rate (FDR)-adjusted *p*-value and log2-ratio were employed to screen the DEGs (Benjamini et al., 2001). The criteria of a two-fold FDR (FDR) <0.05 and an absolute value of log2-ratio > 1 were chosen to determine the significance of up and down-regulated genes.

### GO and Pathway Enrichment Analysis for DEGs

In gene expression profiling analysis, DEGs were mapped to the terms of GO database, to obtain the annotation of GO functional classification and enrichment analysis measured in *p*-value (Xu et al., 2013). Kyoto Encyclopedia of Genes and Genomes (KEGG) were chosen to perform pathway associated with metabolic or signal transduction enrichment analysis of DEGs (Kanehisa et al., 2008).

### RT-PCR and Real-Time Quantitative RT-PCR

Total cell RNA was isolated using Trizol (Invitrogen, United States). The cDNA with oligo (dT) primers were synthesized using the PrimeScript RT reagent Kit (TAKARA, China) with gDNA Eraser, according to the manufacturer’s instructions. Real-time Quantitative RT-PCR was conducted on the iQ5 Multicolor Real-Time PCR Detection System (Bio-Rad, United States), with SYBR *Premix Ex Taq II* (CWBIO, China) reagents, following the manufacturer’s instructions. Sequences of primer pairs used are presented in Supplementary Table S1. The porcine β-actin gene served as a reference gene. Each sample was carried out in technical duplicates. Melting curve analysis and quantitative analysis of the data were performed on iQ5 data analysis software. The relative level of PCV2, GPNMB, ORF5 and Cyclin A were tested using specific primers in Supplementary Table S1. The 2^ΔΔCt^ method was used to analyze relative expression levels of target genes.

### Co-immunoprecipitation (Co-IP) Assays

Exogenous expression and endogenous verification were performed for co-immunoprecipitation (co-IP). For endogenous verification, PAMs co-transfected with 2 μg of pEGFP-ORF5 and 2 μg of Flag-GPNMB plasmids were harvested at 36 h with western blot and IP lysis buffer (Beyotime, China) containing phenylmethanesulfonyl fluoride (PMSF, Solarbio, China). Followed by centrifugation for 30 min at 4°C, a quarter of the supernatant was subjected to input assays. The rest were incubated with Anti-flag M2 affinity gel (Sigma-Aldrich, United States) overnight at 4°C, which had been centrifuged and rinsed with TBS. Followed by washed with TBS and boiled in 5 × SDS sample buffer, the proteins samples were subjected to western blot with mouse anti-GFP monoclonal antibody (mAb) (ZSGB Bio, China) or mouse anti-Flag polyclonal antibody (pAb) (CWBIO, China). For endogenous verification, PAMs transfected with 4 μg of Flag-ORF5 plasmid were harvested, and protein samples were detected with rabbit anti-GPNMB pAb and mouse anti-Flag pAb (Santa Cruz Biotechnology, United States). The other steps were like those in exogenous verification.

### GST Pull-Down Assays

For GST pull-down assays, GST or GST-ORF5 protein was expressed in *Escherichia coli* Rosetta (DE3) cells and Flag-GPNMB protein was expressed in HEK293T cells. The Pierce GST Protein Interaction Pull-Down Kit (Thermo Fisher Scientific, United States) was used according to the manufacturer’s instructions. The proteins produced in *E. coli* were treated with pull-down lysis buffer and then conjugated to glutathione beads glutathione agarose resin for 2 h at 4°C. The beads then were washed with 1:1 wash solution (TBS: Pull-Down Lysis Buffer) for five times and incubated with Flag-GPNMB harvested from HEK293T cells overnight at 4°C. After washing five times, target protein was detected by western blot.

### Western Blot

Cell lysates were prepared in radioimmunoprecipitation (RIPA) buffer with protease inhibitor PMSF and Halt^TM^ phosphatase inhibitor cocktail (Roche, Switzerland). Protein concentrations were confirmed by BCA Protein Assay Reagent (CWBIO, China). Equivalent amounts of protein samples were separated by 12% SDS-PAGE, and the target proteins were transferred to PVDF membranes (Millipore, United States). Membranes were blocked in TBST, a blocking buffer containing 5% skim milk for 2 h at room temperature, followed by incubation with primary antibodies, including rabbit anti-GPNMB polyclonal antibody, rabbit anti-Cyclin A polyclonal antibody (1:1000, Santa Cruz Biotechnology, United States), and rabbit anti-β-actin polyclonal antibody (1:2000, CWBIO, China) at 4°C for overnight. After washes with TBST for five times, membranes were incubated with horseradish peroxidase HRP-conjugated goat anti-rabbit (or mouse) IgG (1:1000, CWBIO, China) for 2 h at room temperature. After washes with TBST for five times, immunoreactive bands were detected using chemiluminescent reagent ECL (Solarbio, China) by GeneGnome XRQ Chemidoc System (Syngene, Cambridge, United Kingdom). The cellular protein β-actin was measured as an internal control.

### Confocal Microscopy

PAMs cultured in glass bottom dishes (35 mm) (Solarbio, China) were co-transfected with pEGFP-ORF5 and PDsRed-GPNMB recombinant plasmids with TurboFect Transfection Reagent (Thermo Fisher Scientific, #R0531). A set of control cells were also subjected to the same experimental conditions. The transfected cells were further cultured for 48 h were rinsed with pre-cooled PBS, then fixed with 4% paraformaldehyde for 20 min at room temperature and washed with PBS buffer for five times, and then stained with 4’, 6-diamidino-2-phenylindole (DAPI) (Solarbio, China) for 5 min. Laser confocal scanning microscopy (Model LSM 510 META, Germany) was used to obtain images.

### Construction of Stable Cell Lines With GPNMB Overexpression or Knockdown

HEK293T cells were cultured in 6-well plates as described above until 70–80% confluent, then were co-transfected with 2 μg pCDH-CMV-MCS-EF1-GreenPuro GPNMB or pCDH-U6-MCS-EF1-GreenPuro-shGPNMB-1, -2 and 3 along with 0.67 μg pGag/Pol, 0.67 μg pRev, and 0.67 μg pVSV-G plasmids. The medium was refreshed with 2 mL advanced DMEM (Gibco, United Kingdom) with 2% FBS, 0.01 mM L-α-phosphatidylcholine, 0.01 mM cholesterol (Sigma-Aldrich, United States), 4.0 mM L-glutamine (Gibco, United Kingdom) and 1:1000 diluted chemically defined lipid (Gibco, United Kingdom) at 16 h post-transfection. Followed by incubation for 48 h, the culture supernatant was collected as lentivirus stock. PK-15 cells or PAMs were transduced with lentiviruses with polybrene to enhance infection rate. The medium was refreshed at 8–10 h post-infection and kept incubation for an additional 48 h. Stable cell lines for GPNMB overexpression or knockdown were seleted by using puromycin at a concentration of 6 μg/mL (Thermo Fisher Scientific, United States). pCDH-CMV-MCS-EF1-GreenPuro (CMV) and random sequence vector (shN) were treated as controls.

### Flow Cytometry Analysis

PAMs stable exhibiting GPNMB overexpression or knockdown were fixed with 75% ethanol 4°C overnight and were then incubated with propidium iodide (PI) in the dark for 0.5 h. A set of control cells was also subjected to the same experimental conditions. The nuclear DNA content was tested by a Coulter Epics XL flow cytometer (Beckman, United States). Data analysis was performed using the CXP Software (Beckman) based on the FSC-SSC (forward light scatter-side scatter) dot plot.

### Statistical Analysis

Statistical analyses were performed on Microsoft Excel and GraphPad Software. Results are presented as mean ± the standard deviations (SD). Student’s *t*-test was used for the statistical comparisons analysis. A *p*-value < 0.05 was considered significant.

## Results

### Quality Evaluation and Analysis of DEGs Libraries

RNA samples from the PCV2-infected and ORF5-transfected PAMs were collected at 48 h post-infection or transfection. Each condition was assayed in triplicate and PAM cell without any treatment was used as mock-control. RNA concentration of Mock, ORF5 and PCV2 were 277, 262, and 157 ng/μL, respectively. The integrity number (RIN) values, yielded from an Agilent 2100 Bioanalyzer (Agilent Technologies) were 9.8, 9.8, and 9.6, respectively, which was an indication of high quality RNA for cDNA library construction and sequencing. The major characteristics of the three cDNA libraries were summarized in Table 1.

**Table 1:** Summary statistics of reads in Mock, PCV2 and ORF5 sample.

Summary	Mock	PCV2	ORF5
Total reads	20959186	21080834	23578294
Filtered reads	20496350	20615300	23070544
Q20	95.68	95.76	95.71
Q30	91.41	91.56	91.43
Unique Mapped Reads	14358732	14336117	14888965
Multiple Mapped Reads	3736350	3784676	4155038
Mapping Ratio	89.17%	88.53%	89.48%
Gene Number	44871	44945	44137
Gene Ratio	89.92%	90.06%	88.45%

*Filtered reads are the remaining reads after filtering out of low-quality reads. Unique Mapped Reads are the reads Unique mapped to the reference sequences. Multiple Mapped Reads are the reads Multiple mapped to the reference sequences. Mapping Ratio = (Unique Mapped Reads + Multiple Mapped Reads)/All Reads Num, Gene Ratio = Gene number in sample/Reference gene number.*

### Screening and Functional Classification of Significantly DEGs

**FIGURE 1:**
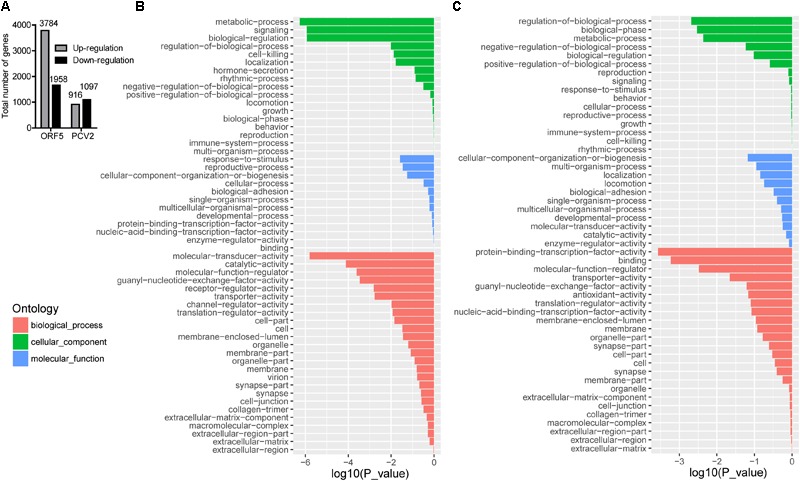
The number of DEGs in different sample groups and GO analysis for up-regulated and down-regulated genes in ORF5 transfected sample. Cell samples were collected at 48 h post-infection or 48 h post-transfection and subjected to transcriptome mapping. The criteria of a two-fold FDR (FDR) < 0.05 and an absolute value of log2-ratio > 1 were chosen to determine the significance of up and down-regulated genes. **(A)** The number of DEGs by pairwise comparison in ORF5 (Mock vs. ORF5) and PCV2 (Mock vs. PCV2) samples. **(B)** GO analysis for up-regulated genes in ORF5 overexpressed PAM cells compared with control. **(C)** GO analysis for down-regulated genes in ORF5 overexpressed PAM cells compared with control. The red, the green and the blue represent biological process, cellular component and molecular function, respectively. The vertical axis represents the GO category, and the horizontal axis represents the negative log values (*p*-values) of the enriched terms.

PCV2 infection and ORF5 transfection stimulated a wide range modification of the host transcriptional profile. As shown in Figure 1A, total DEGs (FDR < 0.05, log2-ratio > 1) were identified by pairwise comparison between different groups (Mock vs. ORF5 and Mock vs. PCV2). Analysis of Go distributions in ORF5 and mock samples showed 54 different patterns of biological processes, cellular components and molecular functions (Figures 1B,C). We also noticed a significant difference in pathways in pairwise comparison in PCV2 and ORF5, including the cell cycle, protein degradation and absorption, various heart-related clinical diseases (Supplementary Figure S1). Notably, the cell cycle pathways were changed in ORF5 transfected cells (Supplementary Figure S2). To analysis the common cellular response between ORF5-overexpressed and PCV2 infected cells, the pathway enrichment analysis was performed. As shown in Supplementary Tables S2, S3, 24 up-regulated and 80 down-regulated genes were both detected in ORF5-transfected and PCV2-infected samples. These genes mainly associated with translation, metabolism, cell cycle, mitogen-activated protein kinases (MAPK) signaling pathways. Interestingly, ORF5 transfection and PCV2 infection down-regulated GPNMB expression level in PAMs, suggesting its potential role in interacting with PCV2 ORF5.

### PCV2 and ORF5 Decreases GPNMB Levels in PAMs

**FIGURE 2:**
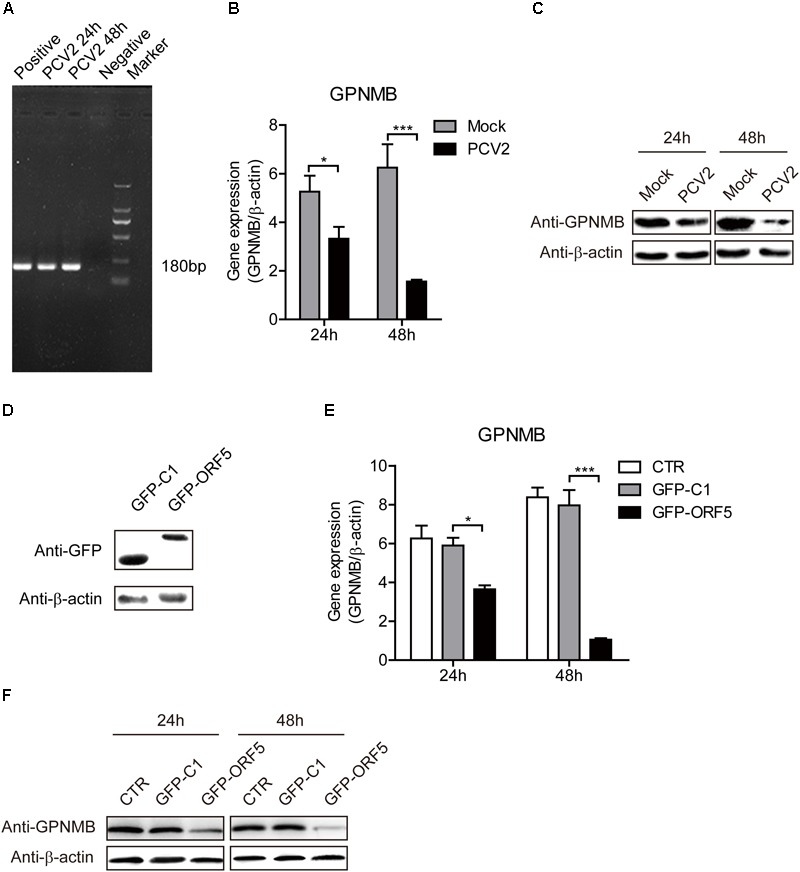
Both PCV2 and ORF5 reduced the expression of GPNMB in PAMs. **(A)** PCR detection of PCV2 nucleic acid in PAM cells with PCV2 infection at a MOI of 1.0 at 24 and 48 h post-infection. Marker. DNA Marker DL1000. **(B)** Real-time qRT-PCR analysis of GPNMB mRNA expression in PAM cells infected with PCV2 a MOI of 1 at indicated time points. **(C)** Immunoblot analysis of GPNMB protein expression in PAM cells infected with PCV2 a MOI of 1 at indicated time points. **(D)** Immunoblot analysis of GFP protein expression in PAM cells transfected with GFP-C1 or GFP ORF5 construct at 48 h post-transfection. **(E)** Real-time qRT-PCR analysis of GPNMB mRNA expression in PAM cells transfected with GFP-C1 or GFP ORF5 construct at indicated time points. **(F)** Immunoblot analysis of GPNMB protein expression in PAM cells transfected with GFP-C1 or GFP ORF5 construct at indicated time points. Data are shown as the mean ± SD of three independent experiments and measured in technical duplicates in **(B)** and **(E)**. Data were normalized to housekeeping gene β-actin expression. Comparisons between groups were determined with the Student’s *t*-test. ^∗^*p* < 0.05; ^∗∗∗^*p* < 0.001.

To further investigate the effect of both PCV2 and ORF5 on GPNMB expression, the GPNMB expression levels was measured in PCV2-infected and GFP-ORF5-transfected cells. The *bona fide* PCV2 infection was confirmed by the detection of PCV2 nucleic acid (Figure 2). As shown in Figures 2B,C, PCV2 infection significantly reduced GPNMB expression at transcriptional and translational levels. To investigate whether PCV2 ORF5 protein lead to the down-regulation of GPNMB, the GFP-fused ORF5 construct was transfected into PAMs and the GPNMB expression was measured at 24 h or 48 h post-transfection (Figure 2D). Consistently, ORF5 overexpression also phenocopying PCV2 infection, which the GPNMB expression was significantly reduced at transcriptional and translational levels (Figures 2E,F). These results suggested that ORF5 is the key viral protein that regulated host GPNMB levels. Taken together, we demonstrated that PCV2 infection down-regulates GPNMB expression and the ORF5 is the key viral protein.

### ORF5 Interacts With GPNMB

**FIGURE 3:**
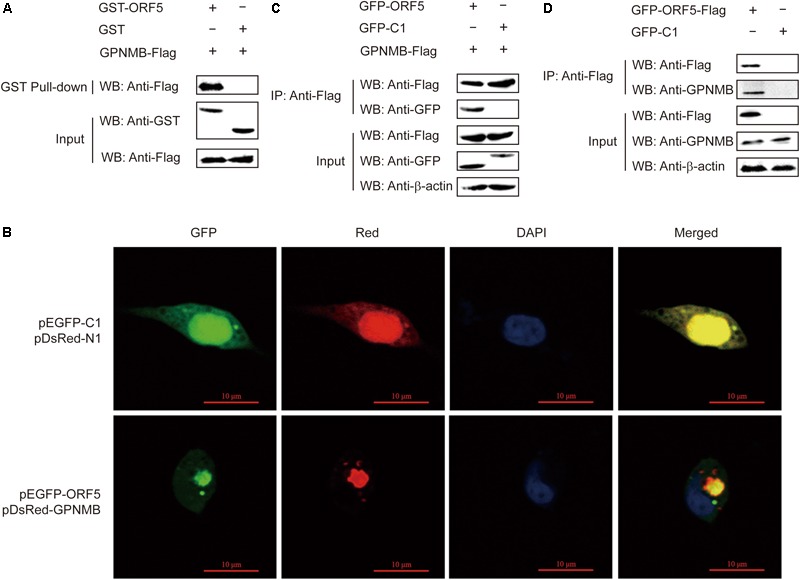
Interaction of ORF5 with GPNMB. **(A)** Exogenous co-IP analysis of ORF5 and GPNMB in PAMs. Cells were co-transfected with plasmids GFP-ORF5 and Flag-GPNMB. PAMs co-transfected with GFP-C1 and Flag-GPNMB were used as negative controls. A quarter of the cell extract was subjected to the input assay to assess β-actin, Flag-fusion and GFP-fusion protein levels. The rest of the extract was subjected to IP assay. Western blot detected proteins with a mouse anti-GFP mAb and a mouse anti-Flag pAb. **(B)** ORF5 protein co-localizes with GPNMB. HEK-293T cells were co-transfected with pGPNMB-Red and pORF5-GFP, pEGFP-C1 and pDsRed-N1 were used as control. Cells were fixed and stained with DAPI (blue) at 48 h post-transfection. Scale bar = 10 μm. **(C)** Endogenous co-IP analysis of ORF5 and GPNMB in PAMs. Cells were transfected with plasmid GFP-ORF5-Flag and GFP-C1-transfected PAMs were used as negative controls. The input assay was performed using a quarter of the cell extract to assess β-actin, Flag fusion protein and GPNMB levels. **(D)** GST-ORF5 pull-down assay. The GST and GST-ORF5 proteins expressed in *Escherichia coli Rosetta* (DE3) cells were immobilized on a glutathione agarose resin, followed by incubation of the resin with the cell lysates containing GPNMB-Flag protein.

We have shown that ORF5 regulates the GPNMB expression in host cells. In our previous study, by using yeast two-hybrid assay, we revealed that ORF5 interacts with GPNMB (Lv et al., 2015). To further elucidate how ORF5 regulates GPNMB expressions, we employed the GST pull-down assay to assess the direct interaction between ORF5 and GPNMB. The GST-fused ORF5 or GST protein were treated with pull-down lysis buffer and then immobilized with glutathione beads, followed by the addition of cell lysates with Flag-GPNMB. The GST pull-down assay clearly showed that the host GPNMB can be captured by GST-ORF5 via a direct physical contact (Figure 3A).

### ORF5 Colocalizes and Interacts With GPNMB in PAMs *in vitro*

To further explore the interaction between GPNMB and ORF5, the confocal fluorescence microscopy was used to determine the cellular localization of GPNMB. PAMs cells were co-transfected with pDsRed-GPNMB and pEGFP-ORF5. As shown in Figure 3B, ORF5 colocalized with GPNMB in the cytoplasm near by the cell nucleus. Next, the co-IP experiments were performed to confirm the direct interaction between GPNMB and ORF5 proteins *in vitro*. The results showed that Flag-GPNMB could co-precipitate with GFP-ORF5 (Figure 3C). In addition, the co-IP assay also revealed the interactions between GPNMB and Flag-ORF5 (Figure 3D). This further confirmed that ORF5 interacts with GPNMB. Taken together, these results demonstrated that ORF5 protein colocalizes and interacts with GPNMB in PAM cells.

### GPNMB Overexpression Inhibits PCV2 Replication and ORF5 Expression

**FIGURE 4:**
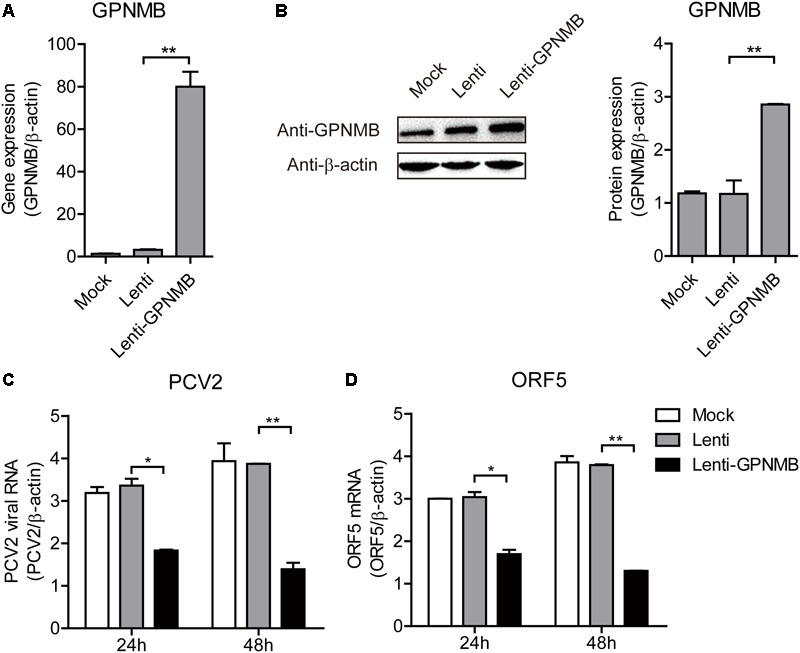
Stable overexpression of GPNMB inhibits PCV2 replication and ORF5 expression. **(A)** Real-time qRT-PCR analysis of GPNMB mRNA expression in PK-15 cells with stable GPNMB overexpression. **(B)** Immunoblot analysis of GPNMB protein in PK-15 cells with stable GPNMB overexpression. The right panel is the GPNMB protein level that the intensity of the signal for targeted protein were normalized to that from β-actin with three independent experiments. **(C,D)** Real-time qRT-PCR analysis of PCV2 viral RNA **(C)** and ORF5 mRNA **(D)** expression in PK-15 cells with stable GPNMB overexpression. Different cell lines were infected with PCV2 at a MOI of 0.1. PCV2 viral RNA level was measured 24 h or 48 h post-infection. Data are shown as the mean ± SD of three independent experiments and measured in technical duplicates in panels **A**, **C**, and **D**. Data were normalized to housekeeping gene β-actin expression. Comparisons between groups were determined with the Student’s *t*-test. ^∗^*p* < 0.05; ^∗∗^*p* < 0.01.

**FIGURE 5:**
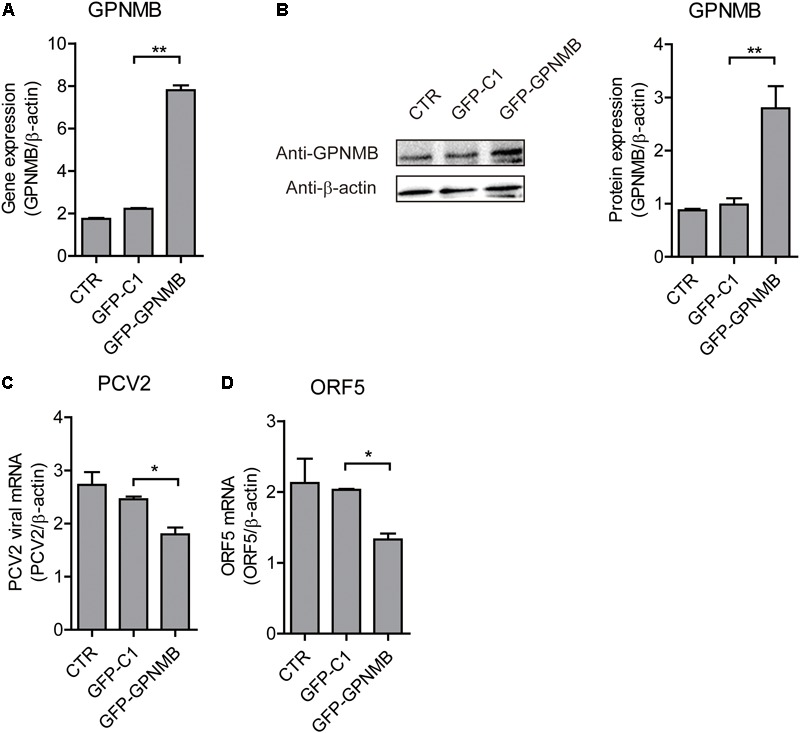
Transient overexpression of Red-fused GPNMB inhibits PCV2 replication and ORF5 expression. **(A)** Real-time qRT-PCR analysis of GPNMB mRNA expression in PK-15 cells transfected with pDsRed-GPNMB or PdsRed-N1 at 24 h post-transfection. **(B)** Immunoblot analysis of GPNMB protein in PK-15 cells transfected with pDsRed-GPNMB or PdsRed-N1 at 24 h post-transfection. The right panel is the GPNMB protein level that the intensity of the signal for targeted protein were normalized to that from β-actin with three independent experiments. **(C,D)** Real-time qRT-PCR analysis of PCV2 viral RNA **(C)** and ORF5 mRNA **(D)** expression in PK-15 cells transfected with pDsRed-GPNMB or PdsRed-N1. Different cells were infected with PCV2 at a MOI of 0.1 at 24 h post-transfection. RNA expression level was measured at 24 h post-infection. Data are shown as the mean ± SD of three independent experiments and measured in technical duplicates in panels **A**, **C**, and **D**. Data were normalized to housekeeping gene β-actin expression. Comparisons between groups were determined with the Student’s *t*-test. ^∗^*p* < 0.05; ^∗∗^*p* < 0.01.

We have shown that PCV2 infection and ORF5 transfection down-regulates GPNMB expression. However, the role of GPNMB in regulating PCV2 replication is still unknown. To determine whether host GPNMB affects PCV2 replication, a PK-15 cell line that stable overexpressing GPNMB (Lenti-GPNMB) was generated. The overexpression of GPNMB was confirmed both at transcriptional and translational levels (Figures 4A,B). The GPNMB-overexpressed cells were infected with PCV2 at a MOI of 0.1. In GPNMB-overexpressed cells, PCV2 genome and ORF5 RNA expression were significantly decreased at 24 and 48 h post-infection, as compared with control (Figures 4C,D). These results indicated that GPNMB overexpression could reduce PCV2 replication and ORF5 expression. To further confirm this observation, PK-15 cells that transfected with GFP-GPNMB plasmid were also infected with PCV2 at a MOI of 0.1. The overexpression of GPNMB was confirmed at transcriptional and translational levels (Figures 5A,B). Consistently, the PCV2 genome and ORF5 RNA expression were also significantly decreased in GFP-GPNMB transfected cells (Figures 5C,D). Together, these results indicated that GPNMB inhibits PCV2 replication and ORF5 expression.

### GPNMB Knockdown Promotes PCV2 Replication and ORF5 Expression

**FIGURE 6:**
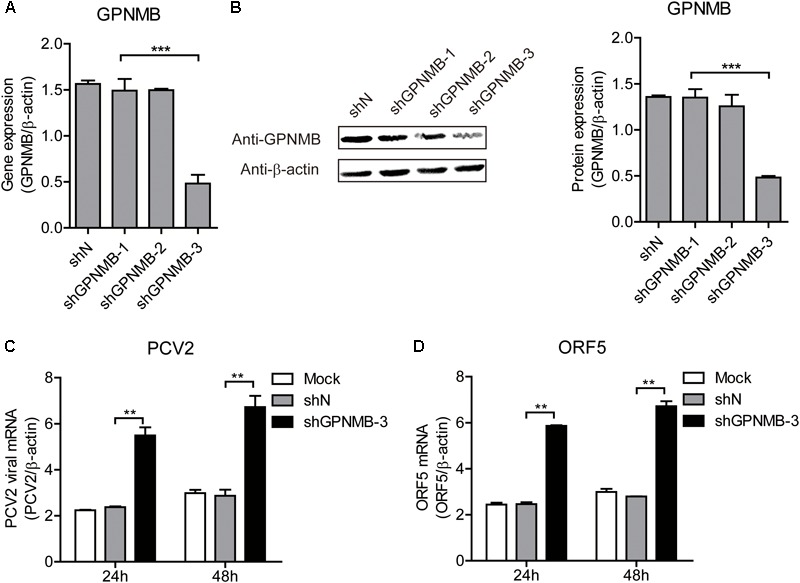
Knockdown of GPNMB increases PCV2 replication and ORF5 expression. **(A)** Real-time qRT-PCR analysis of GPNMB mRNA expression in stable GPNMB knockdown PK-15 cells. PK-15 cells transduced with GPNMB-knockdown lentivirus (shGPNMB-1, 2, 3) or random sequence control (shN). **(B)** Immunoblot analysis of GPNMB protein in stable GPNMB knockdown PK-15 cells. The right panel is the GPNMB protein level that the intensity of the signal for targeted protein were normalized to that from β-actin with three independent experiments. **(C,D)** Real-time qRT-PCR analysis of PCV2 viral RNA **(C)** and ORF5 mRNA **(D)** expression in stable GPNMB knockdown. Different cells were infected with PCV2 at a MOI of 0.1. RNA expression level was measured at 24 h or 48 h post-infection. Data are shown as the mean ± SD of three independent experiments and measured in technical duplicates in panels **A**, **C**, and **D**. Data were normalized to housekeeping gene β-actin expression. Comparisons between groups were determined with the Student’s *t*-test. ^∗∗^*p* < 0.01; ^∗∗∗^*p* < 0.001.

We have revealed that overexpression of GPNMB reduces PCV2 replication and ORF5 expression. To further investigate the anti-PCV2 potential of GPNMB, the GPNMB knockdown PK-15 cells (shGPNMB-1, shGPNMB-2, and shGPNMB-3) were generated. As shown in Figures 6A,B, the highest knockdown efficiency was noticed in the shGPNMB-3 cells and thus this was used in following study. The shN and shGPNMB-3 cells were infected with PCV2 at a MOI of 0.1. At 24 and 48 h post-infection, the PCV2 viral mRNA and ORF5 RNA were significantly increased, compared with that in the control (Figures 6C,D). These results indicated that the PCV2 replication level was higher in GPNMB deficient cells. Together, these data collectively suggested that GPNMB plays vital roles in restricting PCV2 replication.

### GPNMB Participate in the Regulation of Cell Cycle at S Phase

**FIGURE 7:**
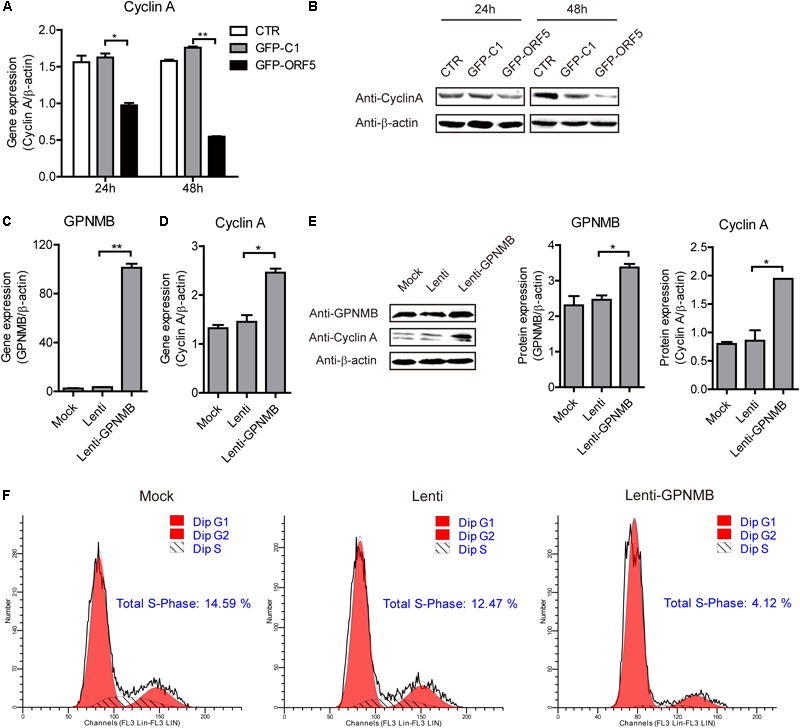
Overexpression of GPNMB promotes cell cycle progression. **(A)** Real-time qRT-PCR analysis of Cyclin A mRNA expression in PAM cells transfected with pEGFP-C1 or pEGFP-ORF5 construct at 24 h or 48 h post-transfection. **(B)** Immunoblot analysis of Cyclin A protein expression in PAM cells transfected with pEGFP-C1 or pEGFP-ORF5 construct at 24 h or 48 h post-transfection. **(C,D)** Real-time qRT-PCR analysis of GPNMB **(C)** and Cyclin A **(D)** mRNA expression in PAMs with stable GPNMB overexpression. **(E)** Immunoblot analysis of GPNMB and Cyclin A protein levels in PAM cells with stable GPNMB overexpression. The middle panel is the GPNMB protein level and the right panel is the Cyclin A protein level that the intensity of the signal for targeted protein were normalized to that from β-actin with three independent experiments. **(F)** Histograms from flow cytometry data for propidium iodide (PI) staining for the mock cells (Mock), control lentivirus transduced cells (Lenti) and GPNMB overexpression lentivirus transduced cells (Lenti-GPNMB). Data are shown as the mean ± SD of three independent experiments and measured in technical duplicates in panels **A** and **B**. Data were normalized to housekeeping gene β-actin expression. Comparisons between groups were determined with the Student’s *t*-test. ^∗^*p* < 0.05; ^∗∗^*p* < 0.01.

It has been demonstrated that the PCV2 replication level is correlated with Cyclin A expression level (Tang et al., 2013). In the present study, the high-throughput sequencing results revealed that the cell cycle pathways were changed in the ORF5-overexpressed cells (Supplementary Figure S2). In our previous study, we have revealed that PCV2 ORF5 protein down-regulate Cyclin A expression and this was also confirmed in the present study (Figures 7A,B) (Lv et al., 2015). However, whether the GPNMB could regulate Cyclin A expression was still unknown. To explore this, we measured the Cyclin A level in GPNMB overexpression or knockdown cells. As shown in Figures 7C–E, overexpression of GPNMB significantly boosted Cyclin A expression. Cyclin A controls G1/S, S/G2, and G2/M phase cell cycle transitions, which are critical for initiation and progression of DNA synthesis (Yam et al., 2002). Thus, we measured the cell cycle in GPNMB-overexpressed cells. As shown in Figure 7F, the proportion of GPNMB-overexpressed cells found to be in S-phase was 4.12%, whereas which for control cells were 14.59% or 12.47%, respectively. The results suggested that the overexpression of GPNMB could trigger a less proportion of cells to enter S-phase.

**FIGURE 8:**
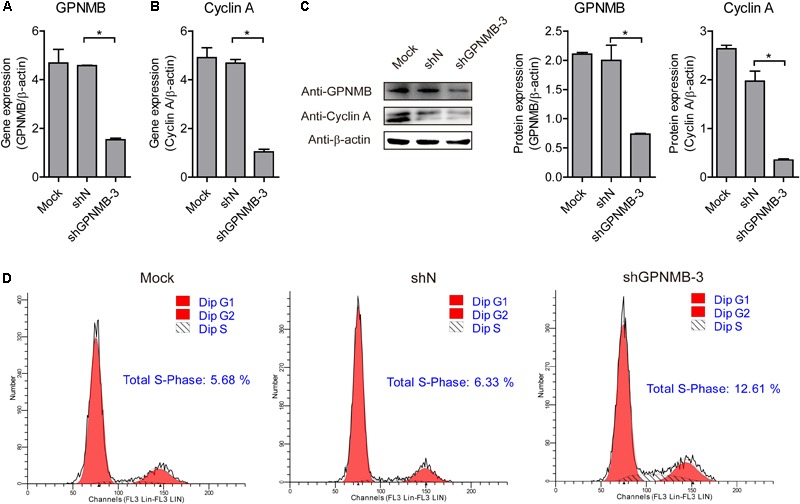
Knockdown of GPNMB inhibits cell cycle progression. **(A,B)** Real-time qRT-PCR analysis of GPNMB mRNA **(A)** and Cyclin A mRNA **(B)** expression in PAMs cell lines with GPNMB knockdown. **(C)** Immunoblot analysis of GPNMB and Cyclin A protein levels in PAM cells with lines with GPNMB knockdown. The middle panel is the GPNMB protein level and the right panel is the Cyclin A protein level that the intensity of the signal for targeted protein were normalized to that from β-actin with three independent experiments. **(D)** Histograms from flow cytometry data for propidium iodide (PI) staining for the mock cells (Mock), control lentivirus transduced cells (shN) and GPNMB knockdown lentivirus transduced cells (shGPNMB-3). Data are shown as the mean ± SD of three independent experiments and measured in technical duplicates in panels **A** and **B**. Data were normalized to housekeeping gene β-actin expression. Comparisons between groups were determined with the Student’s *t*-test. ^∗^*p* < 0.05.

To further confirm that GPNMB regulates Cyclin A expression, we also tested Cyclin A expression in GPNMB knockdown cells. As shown in Figures 8A–C, down-regulation of GPNMB significantly inhibited Cyclin A expression. Consistently, knockdown of GPNMB lead to more cells to enter S-phase (Figure 8D). These results demonstrated that GPNMB positively regulates Cyclin A expression. Together with the observation that GPNMB inhibits PCV2 replication (Figures 4–6) and Cyclin A overexpression suppresses PCV2 replication (Tang et al., 2013), our finding implies that GPNMB inhibits PCV2 infection may through the up-regulation of Cyclin A.

## Discussion

PCV2-associated disease (PCVAD) affects most pig-producing regions and causes massive economic lose (Matzinger et al., 2016). Increasing studies have shown that PCV2 subverts host cellular signals and pathways to benefit its replication. Previous study has revealed that PCV2 ORF3 protein can facilitate the proteasomal degradation of regulator of G protein signaling 16 (RGS16) to promote IL-6 and IL-8 secretion (Choi et al., 2015). Moreover, ORF3 protein also has been reported competes with p53 in binding to Pirh2 and mediates the deregulation of p53 homeostasis (Karuppannan et al., 2010). Additionally, PCV2 ORF4 has been found for stabilizing the concentration of ferritin heavy chain (FHC) to antagonizes host cell apoptosis (Lv et al., 2016). However, whether PCV2 ORF5 is involved in these responses was still unknown. In the present study, the high-throughput sequencing was conducted and the functional classification and pathway enrichment analysis of DEGs were performed to elucidate this enigma. DEGs libraries analysis provided data regarding transcriptomic changes in PAM cells transfected with ORF5 or infected with PCV2. We found more genes were changed in the ORF5-transfected cell compared with PCV2 infected cells. This could be explained by the fact that the transfection efficiency is higher that PCV infection.

In previous study, by using yeast two-hybrid assay, we have identified five proteins potentially interact with ORF5, including GPNMB, CYP1A1, YWHAB, ZNF511, and SRSF3 (Lv et al., 2015). In this study, the transcription analysis of cellular response to PCV2 infection and ORF5-transfection in PAM cells also revealed that the GPNMB expression was affected by PCV2 infection (Supplementary Tables S2, S3). Next, we experimentally proved that PCV2 infection and ORF5 transfection down-regulates GPNMB expression (Figure 2). This is consistent with the transcription analysis done by other group, which also showed that the GPNMB mRNA level was down-regulated after PCV2 infection (Li et al., 2013). The GPNMB was an ORF5-interacting host factor as identified by yeast two-hybrid assay. In this study, we employed diffident approach to validate the interaction between ORF5 and GPNMB. GST-pulldown assay demonstrated that GPNMB can bind to ORF5 protein (Figure 3A). The isolation of Flag-GPNMB/pEGFP-ORF5 or Flag-ORF5/GPNMB complexes suggested that GPNMB could bind to ORF5 *in vitro* (Figures 3C,D). Furthermore, confocal microscopy confirmed the co-localization of GPNMB and ORF5 protein, confirming the interaction between GPNMB and ORF5 (Figure 3B). Taken together, these results convincingly demonstrated that PCV2 ORF5 interacts with host protein GPNMB.

It has been demonstrated that PCV2 infection affects GPNMB expression, but whether the GPNMB can alter PCV2 replication remains unknown. In the present study, the effect of GPNMB on PCV2 replication and ORF5 protein expression were investigated. To this aim, cell lines that stalely overexpressing or knockdown GPNMB were generated. Several results collectively showed that GPNMB inhibited PCV2 replication and ORF5 protein expression (Figure 4). Furthermore, the transient overexpression of GPNMB also inhibited PCV2 replication (Figure 5). Taken together, all these results demonstrated that GPNMB is an important cellular factor that restricts PCV2 replication. Growing evidence identified GPNMB as an attractive therapeutic target for tumor and cancer (Swanton and Jones, 2001; He et al., 2002; Laichalk and Thorley-Lawson, 2005; Grey et al., 2010; Balistreri et al., 2016). Here, we showed the GPNMB plays vital role in restricting PCV2 replication. However, whether the GPNMB could also inhibit other viruses needs further investigations.

It has been generally accepted that viruses could regulate host cellular life cycle to favor their replication (Tischer et al., 1987). Rotavirus replication correlated with S/G2 interphase arrest in cell cycle (Gluck et al., 2017). Herpesviruses (Flemington, 2001), severe acute respiratory syndrome coronavirus (SARS-CoV) protein (Yuan et al., 2005), influenza A virus and its NS1 protein (Jiang et al., 2013), human respiratory syncytial virus and murine norovirus (MNV) (Davies et al., 2015) can induce cell cycle arrest in the G0/G1 phase. PCV2 replication is both S-and G2/M-phase dependent, PCV2 infection induces cellular S phase accumulation via the suppression of Cyclin A (Tang et al., 2013). In addition to the finding that the GPNMB interacted with PCV2 ORF5 and restricted PCV2 replication, we surprisingly find that GPNMB enhanced Cyclin A expression and triggered a less proportion of cells to enter S-phase (Figures 7, 8). This is consistent with the previous study that knockdown of GPNMB suppresses the cell proliferation (Oyewumi et al., 2016). Thus, it reasonable to speculate that the GPNMB inhibits PCV2 infection through the up-regulation of Cyclin A. However, the molecular mechanism still needs further investigations. In addition, whether the PCV2 infection regulates Cyclin A expression via the down-regulation of GPNMB should be dissected in further studies.

## Conclusion

In conclusion, in this study we characterized the GPNMB was down-regulated in PCV2-infected and ORF5 transfected PAM cells by using high-throughput sequencing. Further studies confirmed that PCV2 and ORF5 decreased GPNMB expression and there was direct interaction between ORF5 and GPNMB. In addition, we also revealed that GPNMB restricts PCV2 replication, regulates Cyclin A expression and leads to a less proportion of cells enter S-phase. This study identified a novel host factor GPNMB that interacts with PCV2 ORF5 protein and restricts PCV2 replication and may provide insights into the PCV2-host interaction.

## Author Contributions

KG, LX, MW, and YZ designed the research. KG, MW, YH, YJ, JL, PX, ZF, and RZ performed the research. KG, LX, FX, and YZ analyzed the data. FX and YZ contributed to new reagents and analytic tools. KG, LX, MW, FX, and YZ wrote the manuscript.

## Conflict of Interest Statement

The authors declare that the research was conducted in the absence of any commercial or financial relationships that could be construed as a potential conflict of interest.

## Footnotes

1 http://rnaidesigner.thermofisher.com/

2 https://github.com/BGI-flexlab/SOAPnuke
