# Supplementary material for: A Host Factor GPNMB Restricts Porcine Circovirus Type 2 (PCV2) Replication and Interacts With PCV2 ORF5 Protein
Source: Front Microbiol. 2019 Jan 8;9:3295. doi: 10.3389/fmicb.2018.03295 (PMC6331448; doi:10.3389/fmicb.2018.03295)
Supplement: Supplementary file 1 [file Data_Sheet_1.pdf]

Supplemental Information

A Host Factor GPNMB Restricts Porcine Circovirus Type 2 (PCV2) Replication and Interacts with PCV2 ORF5 Protein

Kangkang Guo, Lei Xu, Mengmeng Wu, Yufeng Hou, Yanfen Jiang, Jiangman Lv, Panpan Xu, Zhixin Fan, Ruiqi Zhang, Fushan Xing, Yanming Zhang

A Host Factor GPNMB Restricts Porcine Circovirus Type 2 (PCV2) Replication and Interacts with PCV2 ORF5 Protein

Kangkang Guo[1#], Lei Xu[2#], Mengmeng Wu[1#], Yufeng Hou[1], Yanfen Jiang[1], Jiangman Lv[1], Panpan Xu[1], Zhixin Fan[1], Ruiqi Zhang[1], Fushan Xing*, Yanming Zhang*

[1]College of Veterinary Medicine, Northwest A&F University, Yangling, Shaanxi 712100, China
[2]College of Life Sciences, Northwest A&F University, Yangling, Shaanxi 712100, China
[#]These authors contributed equally to this article

*Address correspondence to:
Yanming Zhang, Ph.D. Professor
College of Veterinary Medicine, Northwest A&F University
No. 22 Xinong Road, Yangling, Shaanxi 712100, China.
Tel: +86-29-87092040
Email: zhangym@nwsuaf.edu.cn
Fushan Xing, Ph.D.
College of Veterinary Medicine
Northwest A&F University,
No. 22 Xinong Road, Yangling, Shaanxi, 712100, China
Email: xingfushan@nwsuaf.edu.cn

Running title: GPNMB restricts PCV2 and interacts with ORF5

Supplemental Figures

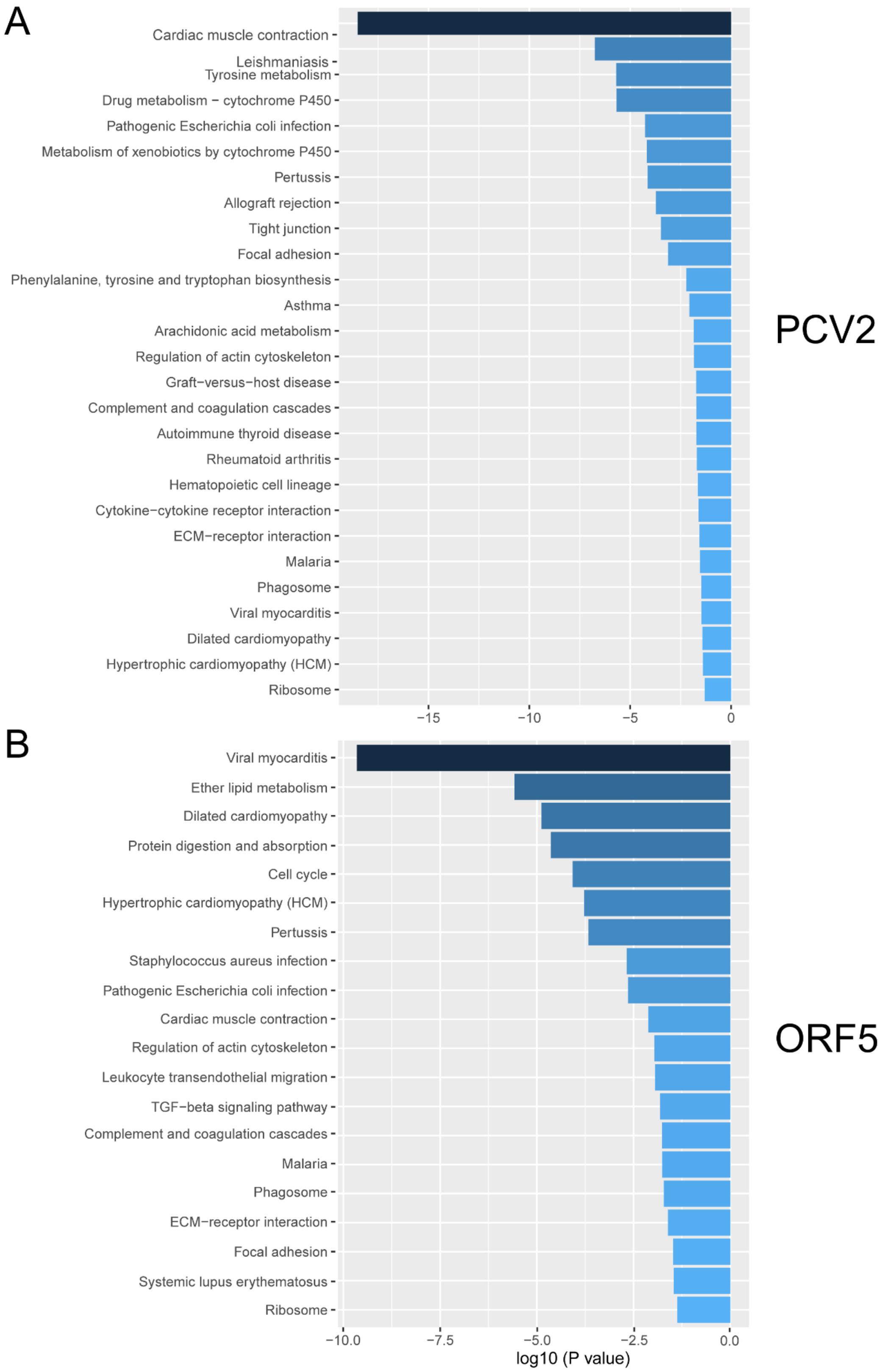

Figure S1. Pathway enrichment analysis for DEGs in ORF5-overexpressed and PCV2-infected PAM cells.

(A) Pathway enrichment analysis for DEGs in PCV2-infected PAM cells compared with control
(B) Pathway enrichment analysis for DEGs in ORF5-overexpressed PAM cells compared with control.
The vertical axis represents the pathway category, and the horizontal axis represents the negative log values (p-values) mapped to each pathway.

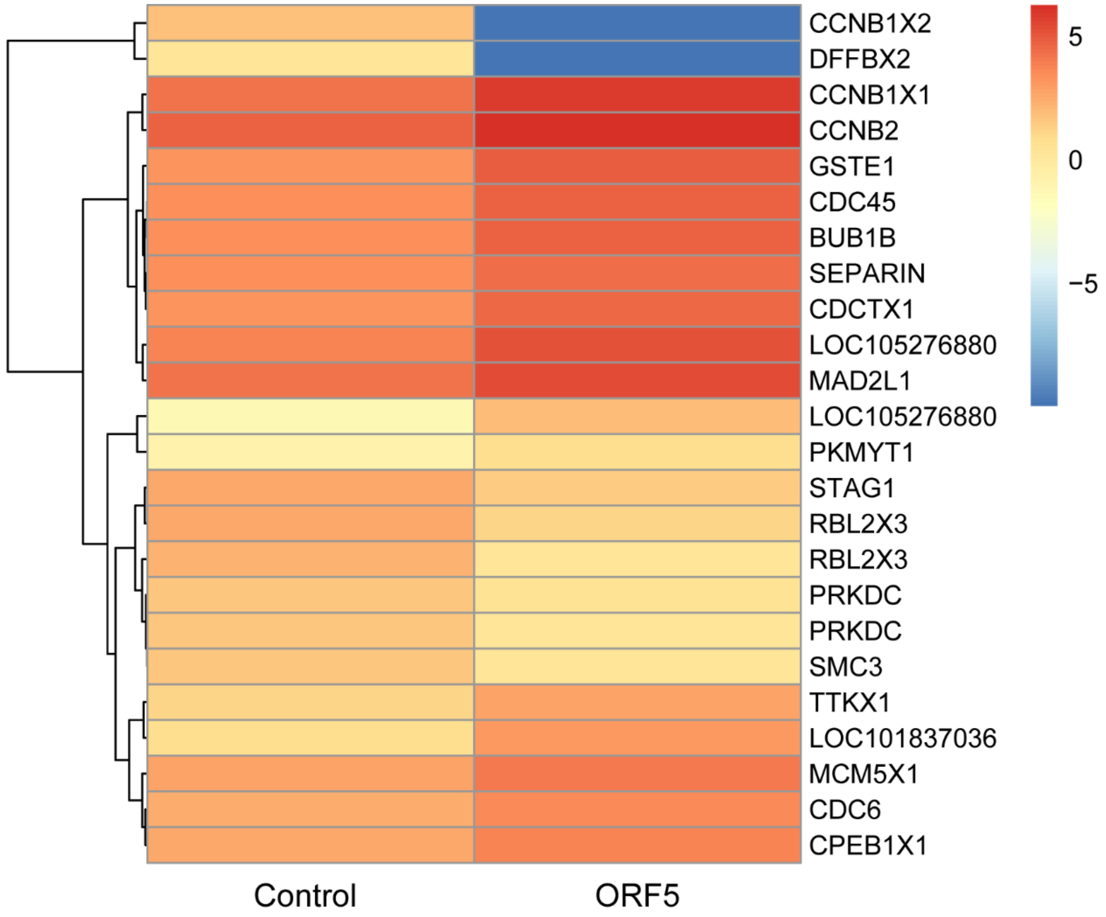

Figure S2. DEGs in ORF5-overexpressed PAM cells.

DEGs in ORF5-overexpressed PAM cells reveals clusters of regulated genes enriched in cell cycle pathways with different response levels. The DEGs significant associated with cell cycle pathway in ORF5-overexpressed PAM cells were annotated and compared with those obtained from control groups.

Supplemental Tables

Table 1. Sequences of primer pairs used for qPCR and plasmid construction.

Primer	Sequence (5'-3')	Use
pDsRed-GPNMB-F	CCGCTCGAGATGGAATGTCTCTACTGTTTTCTGGG	To generate pDsRed-GPNMB
pDsRed-GPNMB-R	CCGGGCCCGGTTCTTGAGCAGTGGATCTTTCTCC	
FLAG-GPNMB-FLAG-F	GCTCTAGATGGATTACAAGGATGACGACGATAAGGAATGTCTCTACTGTTTTCTGGG	To generate FLAG-GPNMB
FLAG-GPNMB-FLAG-R	GCTTCGAATCACTTATCGTCGTCATCCTTGTAATCGTTCTTGAGCAGTGGATCTTTCTCC	
FLAG-ORF5-F	CCGCTCGAGCTATGGATTACAAGGATGACGACGATAAGTACACGTCATTGTGGGG	To generate FLAG-ORF5
FLAG-ORF5-R	CGGGATCCTCACTTATCGTCGTCATCCTTGTAATCGTAGATCATCCCAGGGCAGC	
GST-ORF5-F	CGGGATCCATGTACACGTCATTGTGGGG	To generate pGEX-6P-1-ORF5
GST-ORF5-R	GGCCTCGAGTCAGTAGATCATCCCAGGGCAGC	
pCMV-GPNMB-F	GCTCTAGAATGGAATGTCTCTACTGTTTTCTGGG	To generate CMV-GPNMB
pCMV-GPNMB-R	GCTTCGAATCAGTTCTTGAGCAGTGGATCTTTCTCC	
pEGFP-GPNMB-F	CGGAATTCATGGAATGTCTCTACTGTTTTCTGGG	To generate pEGFP-GPNMB
pEGFP-GPNMB-R	ATTGGATCCGGTTCTTGAGCAGTGGATCTTTCTCC	
PCV2-F	ATGTACACGTCATTGTGGGG	qPCR for detection of PCV2
PCV2-R	TCAGTAGATCATCCCAGGGC	
ORF5-F	ATGTACACGTCATTGTGGGG	qPCR detection of ORF5
ORF5-R	TCAGTAGATCATCCCAGGGC	
GPNMB-F	GGTGACACTTGCTTGCTGAC	qPCR for detection of GPNMB
GPNMB-R	CCGGGGATAGAGACAAGGGT	
Cyclin A-F	CCGGGGATAGAGACAAGGGT	qPCR for detection of Cyclin

Cyclin A-R	GTTCCTCCTTGGAAAGCAAACA	A
β-actin-F	CAAGGACCTCTACGCCAACAC	qPCR for detection of β-actin
β-actin-R	TGGAGGCGCGATGATCTT	
GPNMB-sh1-S	GATCCGGATGTGCTGAGCAATGAAAGCAAGAGCTTTCATTGCTCAGCACATCCTTTTTG	To generate GPNMB sh1
GPNMB-sh1-A	AATTCAAAAAGGATGTGCTGAGCAATGAAAGCTCTTGCTTTCATTGCTCAGCACATCCG	
GPNMB-sh-2-S	GATCCGGGATAATACTGGTCTGTTTGCAAGAGCAAACAGACCAGTATTATCCCTTTTTG	To generate GPNMB sh2
GPNMB-sh2-A	AATTCAAAAAGGGATAATACTGGTCTGTTTGCTCTTGCAAACAGACCAGTATTATCCCG	
GPNMB-sh3-S	GATCCGCGGGAATTCTACAAGTTAACCAAGAGGTTAACTTGTAGAATTCCCGCTTTTTG	To generate GPNMB sh3
GPNMB-sh3-A	AATTCAAAAAGCGGGAATTCTACAAGTTAACCTCTTGGTTAACTTGTAGAATTCCCGCG	
GPNMB-shN-S	GATCCGCTTAAACGCATAGTAGGACTCAAGAGAGTCCTACTATGCGTTTAAGCTTTTTG	To generate GPNMB shN
GPNMB-shN-A	AATTCAAAAAGCTTAAACGCATAGTAGGACTCTCTTGAGTCCTACTATGCGTTTAAGCG	

*Underlined sites indicate restriction enzyme cutting sites added for cloning. Box sites indicate Flag tag.

Table S2. Functional classification of genes common up-regulated significantly in PCV2-infeced and ORF5-overexpressed PAM cells.

Functional classification	Gene name	Gene symbol	log2 Ratio	FDR
Metabolism				
	thyroid hormone receptor beta	THRB	1.16	0.0413
	glutathione S-transferase A2	GSTA2	1.11	0.0287
	glucokinase	GCK	1.96	5.74e-6
	cytosolic phospholipase A2 epsilon isoform X2	PLA2G4E	2.96	2.78e-9
	thiosulfate sulfurtransferase	TST	1.21	0.0002
	uridine phosphorylase 1	UPP1	2.21	2.08e-10
	aconitate hydratase	ACO1	2.06	2.41e-5
Cell cycle				
	wee1-like protein kinase metal ion binding	WEE1	2.26	3.68e-6
	ganglioside GM2 activator	GM2A	1.04	0.0408
	peroxisomal membrane protein 11C isoform X1	PEX11G-X1	1.24	0.0002
	tubulin alpha-8 chain isoform X3	TUBA8-X3	2.19	0.0002
Genetic Information				
	transcription elongation factor B polypeptide 2	TCEB2	2.19	0.0002
	40S ribosomal protein S23-like	RPS23L	2.26	0.0344
	Sptbn4 protein	Sptbn4	1.36	1.04e-5
	serine--tRNA ligase, cytoplasmic-like	SARS	1.26	0.0269
MAPK signaling pathway				
	DNA damage-inducible transcript 3 protein	DDIT3	3.49	6.03e-152
	fibroblast growth factor receptor 1 isoform X8	FGFR1	2.05	0.0036
	amphiregulin	AREG	3.06	3.07e-18
Inflammatory response				
	interleukin-6 cytokine receptor	IL6	1.75	2.15e-5

	binding			
	integrin beta-2	ITGB2	10.38	0.0061
Human Diseases				
	proto-oncogene c-Fos	c-fos	2.13	5.5e-9
	histone H2B type 1-H	HIST1H2BH	3.51	1.25e-93
	histone cluster 1, H3g protein	HIST1H3G	2.60	0.0055
	emerin	EMD	3.26	0.0010

The DEGs are presented with putative function assigned by the Go term and manual annotation (FDR＜0.05, log2-ratio＞1).

Table S3. Functional classification of genes common down-regulated significantly in PCV2-infeced and ORF5-overexpressed PAM cells.

Functional classification	Gene name	Gene symbol	log2 Ratio	FDR
Translation				
	ribosomal protein L10 isoform B	RPL10B	-2.74	4.71e-5
	ribosomal protein L10	RPL10	-4.45	0.0001
	40S ribosomal protein S17	RPS17	-4.52	6.13E-5
	rCG23287, isoform CRA_b		-2.69	1.17E-5
	40S ribosomal protein S16-like	RPS16L	-3.65	0.0001
	60S ribosomal protein L10a-like	RPL10AL	-12.51	3.65E-15
	40S ribosomal protein S4, partial	RPS4	-11.99	1.67E-6
	40S ribosomal protein S4	RPS4	-3.06	0.0079
	60S ribosomal protein L3-like	RPL3L	-3.65	0.0179
	60S ribosomal protein L23, partial	RPL23	-12.76	1.63E-10
	60S ribosomal protein L19-like	RPL19L	-3.06	0.0004
	LOW QUALITY PROTEIN: midasin	MDN1	-2.00	0.0129
	60S ribosomal protein L37	RPL37	-3.65	1.46E-9
	L1a protein	L1A	-3.15	0.0048
	60S ribosomal protein L11-like	RPL11L	-2.15	0.0334
	THO complex 2 (predicted), isoform CRA_b	THO2	-1.86	5.19E-6
	60S ribosomal protein L32	RPL32	-1.99	0.0311
	40S ribosomal protein S8	RPS8	-2.81	2.12E-7
	40S ribosomal protein S11	RPS11	-12.01	4.52E-7
	ribosomal protein S3A	RPS3A	-12.22	4.52E-7
	40S ribosomal protein S20	RPS20	-4.15	1.65E-7
	60S acidic ribosomal protein P1-like isoform X1	RPP1LX1	-3.06	3.76E-12
	40S ribosomal protein S12 isoform X1	RPS12X1	-12.17	1.68E-8
	40S ribosomal protein S2	RPS2	-3.43	9.56E-6
	40S ribosomal protein S2	RPS2	-5.82	4.84E-14
	40S ribosomal protein S25-like	RPS25L	-12.86	8.67E-9
	RecName: Full=60S ribosomal	RPL24	-3.97	3.31E-6

	protein L24			
	60S ribosomal protein L27a	RPL27a	-1.99	0.0311
	60S ribosomal protein L3-like	RPL3L	-12.24	0.0002
	60S ribosomal protein L35a	RPL35a	3.58	0.0002
	40S ribosomal protein S9	RPS9	-4.82	2.92E-6
	40S ribosomal protein S15a isoform X2	RPS15aX2	-11.79	0.0034
	60S ribosomal protein L5	RPL5	-11.66	1.17E-5
	RPL5 protein	RPL5	-12.32	6.12E-6
	60S ribosomal protein L32	RPL32	-3.97	3.31E-6
	monoclonal non-specific suppressor factor beta	MNSFβ	-3.56	9.77E-7
	eukaryotic translation elongation factor 1 alpha 1-like	EEF1A1	-3.98	5.83E-62
	O-phosphoseryl-tRNA(Sec) selenium transferase isoform X1	SEPSECS	-1.59	0.0288
	LOW QUALITY PROTEIN: small ubiquitin-related modifier 2-like	SUMO2	-1.94	0.0001
Locomotion				
	--Unigene0012508		-12.07	6.12E-6
	hypothetical rhabdomyosarcoma antigen MU-RMS-40.3		-2.65	3.04E-24
	myosin-3-like, partial	MYH3	-2.09	3.76E-5
	1-phosphatidylinositol-3-phosphate 5-kinase	PIKFYVE	-1.55	0.0023
Genetic Information				
	protein transport protein Sec61 subunit gamma isoform X2	SEC61GX2	-3.23	0.0028
	UDP-glucose:glycoprotein glucosyltransferase 2 isoform X1	UGGT2X1	-1.76	0.0158
	probable E3 ubiquitin-protein ligase HERC1-like, partial	HERC1L	-1.16	0.0007
MAPK signaling pathway				
	Ras GTPase-activating protein 2	RASA2	-2.41	2.09E-7
	as-related C3 botulinum toxin substrate 3	RAC3	-2.65	0.0014
	ras GTPase-activating protein 2	RASA2	-1.81	0.0011

Human Diseases				
	titin	titin	-1.61	1.42E-10
	titin-like protein	Titinl	-1.21	0.0198
	leucine-rich repeat serine/threonine-protein kinase 2	LRRK2	-1.48	5.56E-5
	titin-like protein	titin1	-1.97	0.0022
	troponin T, cardiac muscle	TNNT2	-12.73	5.53E-31
	transcriptional activator GLI3 isoform X1	GLI3X1	-1.91	0.0457
	dynein heavy chain 3, axonemal	DNAH3	-1.50	1.05E-7
Metabolism				
	rCG53488	Rcg53488	-3.52	0.0003
	ATP synthase beta-subunit	ATPSβ	-12.17	0.0003
	Nme2 protein	NME2	-3.67	1.76E-14
	Phosphatidylinositol-4-phosphate 3-kinase C2 domain-containing alpha polypeptide	PIL3C2A	-1.34	0.0016
	ATP synthase subunit beta, mitochondrial, partial	ATP5B	-12.29	3.20E-6
	ATP synthase lipid-binding protein, partial		-3.23	1.47E-6
	canalicular multispecific organic anion transporter 1	G5AYP1	-11.57	0.0110
	arginine kinase, partial	AK	-3.35	9.00E-37
	glyceraldehyde-3-phosphate dehydrogenase,partial	GAPDH	-11.79	0.0005
	glyceraldehyde-3-phosphate dehydrogenase	GAPDH	-3.92	6.00E-6
p53 signaling pathway				
	G2/mitotic-specific cyclin-B1 isoform X2	CCNB1X2	-11.82	4.21E-5
Phagosome				
	tubulin beta-1-like	TUBB1L	-11.91	0.0018
	Tubulin beta-2A chain, partial	TUBB2A	-12.08	0.0003

	tubulin beta-2 chain-like, partial	TUBB2	-11.76	0.0005
Signal transducer activity				
	tenascin-N	TNN	-1.96	7.76E-26
	tenascin-N	TNN	-2.06	4.97e-12
	myosin heavy chain	MHC	-2.31	6.04e-5
	actin, partial structural molecule activity; GO:0008134//transcription factor	actin	-4.06	0.0020
	transmembrane glycoprotein NMB precursor	GPNMB	-2.13	0.0002
	mKIAA1999 protein	mKIAA1999	-1.90	0.0119
	peptidyl-prolyl cis-trans isomerase-like	PPIL	-3.35	2.71E-7
	cartilage: oligomeric: matrix: protein	COMP	-3.78	0
	ADP/ATP translocase 1ribonucleoprotein complex binding		-12.05	2.22E-5
	1-phosphatidylinositol 3-phosphate 5-kinase isoform X7	PIKFYVE	-1.21	0.0485
	thrombospondin-3b-like	LOC105901446	-4.27	3.62E-253

The DEGs are presented with putative function assigned by the Go term and manual annotation (FDR＜0.05, log2-ratio＞1).
